# Migraine in the Young Brain: Adolescents vs. Young Adults

**DOI:** 10.3389/fnhum.2019.00087

**Published:** 2019-03-22

**Authors:** Elisabeth Colon, Allison Ludwick, Sophie L. Wilcox, Andrew M. Youssef, Amy Danehy, Damien A. Fair, Alyssa A. Lebel, Rami Burstein, Lino Becerra, David Borsook

**Affiliations:** ^1^Department of Anesthesiology, Perioperative and Pain Medicine, Center for Pain and the Brain, Boston Children’s Hospital, Harvard Medical School, Boston, MA, United States; ^2^Department of Radiology, Boston Children’s Hospital, Boston, MA, United States; ^3^Department of Behavioral Neuroscience, Oregon Health & Science University, Portland, OR, United States; ^4^Pediatric Headache Program, Department of Anesthesiology, Perioperative and Pain Medicine, Boston Children’s Hospital, Waltham, MA, United States; ^5^Department of Neurology, Boston Children’s Hospital, Waltham, MA, United States; ^6^Department of Anesthesia, Critical Care and Pain Medicine, Beth Israel Deaconess Medical Center, Harvard Medical School, Boston, MA, United States

**Keywords:** pediatric, brain development, functional magnetic resonance imaging, age-related, resting-state functional connectivity, brain, headaches

## Abstract

Migraine is a disease that peaks in late adolescence and early adulthood. The aim of this study was to evaluate age-related brain changes in resting state functional connectivity (rs-FC) in migraineurs vs. age-sex matched healthy controls at two developmental stages: adolescence vs. young adulthood. The effect of the disease was assessed within each developmental group and age- and sex-matched healthy controls and between developmental groups (migraine-related age effects). Globally the within group comparisons indicated more widespread abnormal rs-FC in the adolescents than in the young adults and more abnormal rs-FC associated with sensory networks in the young adults. Direct comparison of the two groups showed a number of significant changes: (1) more connectivity changes in the default mode network in the adolescents than in the young adults; (2) stronger rs-FC in the cerebellum network in the adolescents in comparison to young adults; and (3) stronger rs-FC in the executive and sensorimotor network in the young adults. The duration and frequency of the disease were differently associated with baseline intrinsic connectivity in the two groups. fMRI resting state networks demonstrate significant changes in brain function at critical time point of brain development and that potentially different treatment responsivity for the disease may result.

## Introduction

Migraine is a common disease frequently beginning in childhood, with its highest prevalence in adolescence and early adulthood – prevalence peaks in late teens and early twenties (Victor et al., 2010). The disease presents in different ways with age from childhood to adulthood in various categories, including duration, frequency, or location (Kelman, 2006; Sonal Sekhar et al., 2012). Given that many patients have migraine for many years, the notion of how the disease may show differences in brain function, particularly with respect to neurodevelopment is a critical question. Major neurodevelopmental changes occur from teenage years to the adult brain. Indeed, adolescence is a pivotal period of brain development and maturation that coincides with changes in several domains such as cognitive, emotional, social, and with heightened vulnerability to psychiatric disorders and behavioral problems (Paus et al., 2008). While an extensive literature exits on adolescent neurodevelopment (Casey et al., 2008, 2011; Spear, 2013; Baker et al., 2015), very little information is reported on the potential changes in brain function during this critical period in migraineurs. Differential development of subcortical and cortical processes may provide a basis for different neural network interactions and thus function (Blakemore, 2012; Rubia, 2013; Stevens, 2016), making adolescents and young adults likely to have different responses to the disease and treatments. Indeed, during adolescence, changes in functional connectivity strengths and network relationships are still happening (Marek et al., 2015; Stevens, 2016) and brain differences with young adults include the relative development of subcortical limbic systems vs. top-down control systems and a shift from local to distal connectivity profiles (Power et al., 2010; Wang et al., 2012; Baker et al., 2015; Ernst et al., 2015; Stevens, 2016). One approach to begin to dissect brain related changes within different age cohorts in migraine would be to use longitudinal imaging studies. Yet that kind of approach is challenging, especially in school-age children. Another approach is to evaluate potential similarities and differences in brain resting state functional connectivity (rs-FC) as they may occur with age (viz., childhood vs. adults).

In the past decade, several studies have evaluated the potential alteration of baseline intrinsic brain activity generated by long-term episodes of migraine attacks in adults. These studies showed that brain connectivity at the level of pain processing network, affective network, default mode network (DMN), executive control network (CEN), salience network (SN), and visual network (VN) differ between healthy controls (HC) and migraineurs (Colombo et al., 2015; Coppola et al., 2015; Schulte and May, 2016). In the pediatric population, one can find a few structural and functional neuroimaging data (Rocca et al., 2014; Faria et al., 2015; Youssef et al., 2017; Messina et al., 2018), but only one study that examined seed based rs-FC in early adolescent population. This study reports significantly greater rs-FC in migraineurs in comparison to HC mainly in a few seed regions: (1) the precuneus, which was associated with the DMN and (2) the amygdala and part of the thalamus, which were associated with the SN (Faria et al., 2015). The purpose of the current study was to expand our understanding of how migraine may affect the development of the young brain by studying rs-FC changes at different developmental time points.

In this study, we used an independent component analysis (ICA) coupled with a dual-regression method (Filippini et al., 2009) to examine all the major RSNs in 12–27 years migraineurs compared with age- and sex-matched HC. Age was treated as a categorical variable to assess changes in rs-FC in migraineurs at two developmental stages: adolescence (children from 12 to 18 years) and young adulthood (19–27 years). We also assessed the association between rs-FC patterns within each developmental group and individual migraine characteristics (i.e., duration of migraine in years and attack frequency per month). We hypothesized that (1) In accordance with previous adult and pediatric rs-FC studies performed in migraine and other pain conditions, we expected to find differences in comparison to controls mainly in pain processing network, DMN, SN, CEN, and VN in the young adults group but also in the adolescents group; (2) given stages of brain development (Baker et al., 2015; Zhao et al., 2015), the reported differences in brain function in adolescents vs. young adults (Uddin et al., 2010; Hwang et al., 2013), and evolving maturation of emotional and cognitive processes during adolescence (Paus et al., 2008), we expected to find more differences in baseline intrinsic brain connectivity between adolescents and HC (within comparison) and for the adolescent group in the between comparison [(adolescents > HC) > (young adults > HC)] in networks particularly involved in higher cognitive functions and emotions regulation, i.e., the DMN, SN, CEN, FPN (Menon, 2011); and (3) Finally, we hypothesize that the duration of the disease will be associated with more rs-FC brain changes in young adults with migraine than in adolescents. Some previous studies have highlighted a correlation between changes in brain structure and function and disease duration (Yu et al., 2012; Jin et al., 2013; Xue et al., 2013). This is the first report of RSN changes in migraineurs during this transition state of brain development.

## Materials and Methods

### Ethics Statemant

All procedures performed in studies involving human participants were in accordance with the ethical standards of the institutional and/or national research committee and with the 1964 Declaration of Helsinki and its later amendments or comparable ethical standards. This article does not contain any studies with animals performed by any of the authors.

### Participants

Before the experiment, all participants and parents received a complete description of the study and granted written informed consent. Study participants were drawn from a larger study cohort including 7–27 years-old volunteers with a history of episodic migraine (with or without aura) and age- and sex-matched HC. From this cohort, arterial spin labeling analysis has previously been reported (Youssef et al., 2017). Overall, 51 right-handed adolescents and young adults with migraine and 51 HC were included in this particular study. All patients were recruited from the Neurology and Headache Clinics at Boston Children’s Hospital and advertisements within the general community (Longwood Medical and Greater Boston Area). All patients met the criteria for episodic migraine as defined by the International Classification for Headache Disorders, second edition (ICHD-III)^1^. A study physician confirmed diagnoses during a clinical interview. Age- and sex-matched HC were recruited through: (1) flyers posted on bulletin boards in the Longwood Medical Area and Greater Boston Area; (2) online list servers (e.g., Craigslist, Colleges, job boards); and (3) previous participants in the group’s studies. HC were excluded if they reported symptoms consistent with any type of migraine, or if they had any ongoing pain condition. Patients were excluded if they had continuous background headache and/or were taking prophylactic migraine treatment. All participants (migraine or HC) were excluded if they had significant medical problem (e.g., systemic or CNS, sickle cell anemia, severe psychiatric disorders, and other neurological disorders than migraine), claustrophobia, were pregnant, were left-handed, had metallic implants and/or devices, or weight >235 lbs (magnet table limits). Patients experiencing a migraine within 72 h before the study visit were rescheduled. Study visits included various questionnaires comprising demographic and medical history examination, psychological evaluation, headache specific questionnaires and multimodal MRI. The headache history questionnaire included for example questions regarding age of migraine onset, attack frequency, attack duration, accompanying symptoms (i.e., nausea, vomiting photophobia, phonophobia) and medication usage. Prior to scanning, urine drug screening was performed on all subjects to exclude the possibility of substance abuse. The Institutional Review Board at Boston Children’s Hospital approved the study and the protocol conformed to the latest revision of the Declaration of Helsinki and the International Association for the study of Pain criteria for performing human pain investigations.

### Magnetic Resonance Imaging (MRI) Acquisition

Structural and functional subjects data were acquired on Siemens Magnetom trio 3 Tesla scanner (Siemens Healthcare Inc., United States) equipped with a 32-channel head coil. For image registration, a high-resolution T1-weighted anatomical image was collected using a magnetization-prepared, rapid-acquisition gradient-echo (MPRAGE) (176 slice; slice thickness = 1 mm; TR = 2520 ms; TE = 1.74 ms; TI = 800 ms; FOV = 220 mm^2^, matrix size = 220 × 220). The resting state imaging sequence consisted of a T2^∗^-weighted echo planar imaging sequence (300 volumes, slice thickness = 5 mm, slice number = 34, slice order = interleaved, TR = 2010 ms, TE = 30 ms, flip angle = 90°, FOV = 224 mm^2^, matrix size = 64 × 64, voxel size 3.5 mm × 3.5 mm). Subjects were instructed to relax and to keep their eyes open during the 10 min 5 s of the resting state scan.

### MRI Analysis

#### Preprocessing

The rs-FC analyses were performed with FMRIB’s Software Library (FSL) tools^2^ and the Multivariate Exploratory Linear Optimized Decomposition into Independent Components (MELODIC)^3^. Preprocessing steps on the 4D volume included: (1) motion correction using FMRIB’s Linear Image Registration Tool (MCFLIRT) (Jenkinson et al., 2002); (2) removal of non-brain tissue using FSL’s script Brain Extraction Tool (BET); and (3) high-pass temporal filtering (100 s) to remove slow drift. Subjects were excluded if peak of motion higher than 2 mm or 2° was detected from MCFLIRT estimated rotations, translations, or mean displacement.

Subsequently, single-subject ICA was run in MELODIC on the preprocessed data with variance-normalize timecourses and automatic dimensionality estimation. fMRI volumes were registered to the individual’s structural scan using FMRIB’s Linear Image Registration Tool (FLIRT) which used an automated affine registration algorithm. Then we used FMRIB’s ICA-based Xnoiseifier (FIX) that allows automatically detecting and removing residual noise-related artifacts from fMRI data. FIX removes independent components deemed of no neurological origin such as head motion-related, scanner artifacts, physiological, and other artifacts linked to image acquisition (Griffanti et al., 2014; Salimi-Khorshidi et al., 2014). The algorithm compares the independent components (ICs) to temporal and spatial features extracted from a training dataset and classified subject-level ICA components as “signal” or “noise.” It then denoises the resting-state fMRI data by regressing out time series classified as noise. In the present study we used the “Standard.RData” training data sets that are offered with the standard implementation of FIX (Griffanti et al., 2014; Salimi-Khorshidi et al., 2014) and a threshold of 5.

#### Identification of Resting-State Networks

To investigate group differences in rs-FC within each group of migraine patients (adolescents and young adults) in comparison to HC as well as the migraine-related age differences, an ICA-based approach in combination with a regression technique (dual regression) that allows for voxel-wise comparisons of resting-state connectivity (Beckman et al., 2009; Filippini et al., 2009) was applied on the data. This approach has been widely used to performed between-subject analysis of resting state data in previous papers (Filippini et al., 2009; Uddin et al., 2010; Veer et al., 2010; Mostert et al., 2016; Muetzel et al., 2016). The ICA is a data-driven approach that allows the investigation of functional connectivity in all major resting-state networks (RSNs) without an a-priori selection of a particular region of interest and extracts maximally independent patterns of coherent FMRI activity, which are linearly mixed in the data. Each pattern is composed of a time-course and an associated spatial map and named independent component (Beckmann et al., 2005). Multi-subject ICA and dual regression does not rely on a single seed location but integrates the temporal information in the FMRI data across multiple distributed networks identified in the initial group ICA (Beckmann et al., 2005).

This technique proceeds in three steps. First, a standard group independent component analysis was performed using probabilistic ICA on all the participants (Beckmann et al., 2005) as implemented in MELODIC (CONCAT). A spatial smoothing was performed using a Gaussian kernel of full-width at half maximum (FWHM) of 7-mm and fMRI volumes were registered to the individual’s structural scan and standard space image using the Montreal Neurological Institute-152 and an affine transform with 12° of freedom via FLIRT. Preprocessed functional data were then temporally concatenated across subjects to create a single 4D data set. The concatenated multiple fMRI data sets were then decomposed using ICA to identify global, distinct patterns of functional connectivity in the entire subject population. We limited the number of ICs to 75. We limited the number of ICs to 75. In prior reports 20–30 components have been used and the literature indicates that differences within networks where being collapsed into aggregate networks due to the small number of components chosen (Greicius et al., 2007; Seeley et al., 2007; Uddin et al., 2010, 2013; Veer et al., 2010).

It has been suggested that around 70 components allows to identify differences within networks without approaching an exceedingly fine-grain decomposition of these networks into individual brain structures (Kiviniemi et al., 2009; Abou-Elseoud et al., 2010; Allen et al., 2011). From these 75 ICs, RSNs of interest were selected first by using spatial correlation (Pearson’s r) between the 75 ICS from the group ICA and a set of previously defined resting-state network maps (Smith et al., 2009). Only ICs with Pearson spatial correlation coefficients of at least 0.25 were selected. Second, these selected ICs were visually inspected to confirm the association with previously defined resting-state networks.

Secondly, the set of spatial maps from the group-average analysis was used to generate subject-specific versions of the spatial maps, and associated time-series, using dual regression (see^4^) as described previously (Filippini et al., 2009). This involved using the full set of group-ICA spatial maps in a linear model fit (spatial regression) against the separate individual fMRI data, resulting in matrices describing temporal dynamics for each component and subject (Filippini et al., 2009). The time-course matrices were then entered in a second (temporal) regression against associated data to estimate the 75 spatial component maps for each individual. Thirdly, the different component maps were collected across subjects into single 4D files (one per original ICA map, with the four dimensions being subject identification) and tested for the migraine effect within each developmental group (adolescents and young adults with migraine) and between developmental groups (migraine-related age differences) in comparison to matched HC. It should be noted that we ran one Group ICA on both control and migraine data together.

To evaluate these group differences, we used a group-level GLM analysis (see^5^) and unpaired *t*-test design assessing the following contrasts: (1) Migraine effect in adolescents (adolescents with migraine vs. adolescents HC), (2) Migraine effect in young adults (young adults with migraine vs. young adults HC), (3) Interaction of migraine by age [(adolescents with migraine > HC) > (young adults with migraine > HC); (young adults with migraine > HC) > (adolescents with migraine > HC)]. Although adolescence is typically defined as the developmental stage occurring between puberty and legal adulthood and varies between individuals, no clear standard currently exists for grouping subjects into age band. Consequently, we have roughly followed binning used in previous papers investigating developmental effect on functional network, i.e., adolescence from 12 to 18 years and young adulthood from 19 to 27 years of age (Williams et al., 2006; Kelly et al., 2009; Hwang et al., 2013; Rubia, 2013; Marek et al., 2015).

These analyses result in statistical spatial maps characterizing each contrast. For each of them, statistical parametric maps (SPM) were subjected to alternative hypothesis testing using Gaussian mixture model (GMM) (Pendse et al., 2009). This approach is more accurate in dealing with not normally distributed SPMs as compared to traditional methods by adaptively estimating the form and fraction of “null” from the data (Pendse et al., 2009). We used the mixture model as defined previously^6^. The mixture model was spatially regularized using a Markov random field (MRF) that was soft-max prior on the class labels [Classes are null, significantly anticorrelated and significantly correlated; the rational as for other posterior probability approaches is to assign to a particular class if the voxel’s probability of belonging to that class is more that 50% (P.0.5)]. This prior encourages spatially neighboring voxels to have similar labels. The mixture model parameters as well as the MRF parameter were adaptively estimated from the data using iterated conditional modes (ICM). The posterior probability maps (PPMs) giving the “activation” probability of a voxel conditional on the estimated labels in its neighborhood and the observed data were created and thresholded at PPM >0.5 to detect “activation.”

Thresholds of activation/deactivation maps were then used to determine clusters of activation (peak and volume) using in-house MATLAB programs (MathWorks Inc., Natick, MA, United States). Only clusters of at least 1 cm^3^ (minimum clusters of three smoothed native-space voxels) were considered significant. After clustering, peak activity within each cluster was referred to a standard MRI atlas (Maldjian et al., 2003) and tabulated.

Finally, within each developmental migraine group, we evaluate the linear relation between rs-FC and clinical indicators of migraine: frequency (per month) and duration (in years) of migraine. In each developmental group, a GLM analysis was done with frequency or duration of the migraine as covariate of interest. This analysis was performed using the IC’s selected before and followed by the same procedure of dual-regression.

#### Movement Analysis

To be ascertaining that differences in motion during the scan were not contributing to between-group differences in rs-FC, we compared the single-subject mean relative displacement and absolute displacement (calculated during MCFLIRT motion correction) between migraine patients and HC using unpaired *t*-test. The association between age and movement (relative and absolute displacement) was also tested with Pearson’s correlation in migraine patients and HC.

## Results

### Participants

All participants with migraine were age- and sex-matched with HC. From the 102 right-handed adolescents and young adults included in the study (51 migraineurs and 51 HC), 30 pairs of participants were excluded from the analyses (migraineurs and corresponding HC). Specific exclusions issues included: (1) After quality assurance and pre-processing of the data, 2 HC were excluded due to brain abnormalities and 4 migraine participants due to positive drug testing, headache just before or during the scan, or problem of registration; (2) 2 HC and 3 migraine participants were excluded due to peaks of motion higher than 2 mm or 2 degrees during the scan detected from MCFLIRT estimated rotations, translations, or mean displacement; and (3) to avoid an unbalanced number of males and females in the analysis, 3 more females (2 adolescents and 1 young adult) and one young adult male with migraine were randomly excluded from the analyses.

Among the migraine participants, 2 experienced a migraine 48h following the scanning session (1 adolescent and 1 young adult males), two 72 h following the scanning session (1 adolescent female and 1 young adult male), and one within 72 h following the scanning session (1 adolescent male).

Overall, 36 migraine participants from 12 to 27 years old (mean ± SD age: 19.23 ± 4.45) and their matched HC remained in the analysis. Table 1 summarizes the overall demographic characteristics of participants. There is no difference of age between male and female migraine patients in the adolescent group [mean age (mean ± SD): male adolescents: 15.21 ± 1.67, female adolescents: 15.27 ± 1.78, *t*(16) = -0.07, *p* = 0.95], or in the young adult group [male young adults: 23.57 ± 2.21, females young adults: 22.87 ± 1.99, *t*(16) = 0.71, *p* = 0.49].

**Table 1:** Demographic characteristics of migraine and healthy controls participants.

	Adolescents				Young adults			
	Healthy controls		Migraine patients		Healthy controls		Migraine patients	
	
	Overall				Overall			
N	18		18		18		18	
Age (year) [Mean ± SD (range)]	15.21 ± 1.93 (12–19)		15.24 ± 1.67 (12–18)		22.91 ± 2.08 (20–27)		23.22 ± 2.07 (20–27)	

	**Male**	**Female**	**Male**	**Female**	**Male**	**Female**	**Male**	**Female**

N	9	9	9	9	9	9	9	9
Age (year) (Mean ± SD)	15.04 ± 1.99	15.39 ± 1.96	15.21 ± 1.67	15.27 ± 1.78	23.19 ± 2.42	22.63 ± 1.78	23.57 ± 2.21	22.87 ± 1.99


A summary of migraine patient characteristics by developmental age is presented in Table 2. The mean age at migraine onset and the mean frequency of migraine attacks per month did not differ between adolescents and young adults. In contrast, the mean disease duration in years significantly differed between the two developmental groups [Disease duration (mean ± SD): adolescents: 3.69 ± 2.41, young adults: 9.56 ± 3.87, *t*(34) = -5.46, *p* < 0.001]. There was no difference between the two groups regarding the occurrence of accompanying symptoms of migraine (nausea, vomit, photophobia, phonophobia).

**Table 2:** Migraine patient characteristics by developmental age.

	Adolescents	Young adults	Test statistic	df	*p*
Age of onset	10.88 ± 2.52 (7–17)	12.94 ± 4.52 (7247)	*t* = -1.69	34	*p* = 0.10
Duration (years) [mean ± SD (range)]	3.69 ± 2.41 (0.5–10)	9.56 ± 3.87 (2–16)	*t* = -5.46	34	*p* < 0.001
Attack frequency (month) [mean ± SD (range)]	3.05 ± 3.54 (0.13–14)	2.8 ± 2.57 (0.13–10)	*t* = -0.24	34	*p* = 0.81
Nausea (%)	77.78%	72.22%	χ^2^ = 0.15	1	*p* = 0.7
Vomiting (%)	27.78%	22.22%	χ^2^ = 0.15	1	*p* = 0.7
Photophobia (%)	100%	88.89%	χ^2^ = 2.12	1	*p* = 0.15
Phonophobia (%)	94.12%	72.22%	χ^2^ = 2.95	1	*p* = 0.08


### Movement Analysis

The mean relative displacement and absolute displacement did not differ between the migraine patients and the HC [*Relative displacement* (mean ± SD): migraine patients: 0.06 ± 0.03 mm, HC: 0.06 ± 0.02 mm, *t*(70) = 0.05, *p* = 0.96; *Absolute displacement*: migraine patients: 0.25 ± 0.12, HC: 0.28 ± 0.13, *t*(70) = -1.13, *p* = 0.26], nor between adolescents and young adults with migraine [*Relative displacement*: adolescents migraineurs: 0.07 ± 0.04 mm, young adult migraineurs: 0.06 ± 0.02 mm, *t*(34) = 1.07, *p* = 0.29; *Absolute displacement*: adolescents migraineurs: 0.28 ± 0.13 mm, young adult migraineurs: 0.22 ± 0.10 mm, *t*(34) = 1.47, *p* = 0.15], or between adolescents HC and young adults HC [*Relative displacement*: adolescents: 0.06 ± 0.02, young adults: 0.06 ± 0.02, *t*(34) = -0.45, *p* = 0.66; *Absolute displacement*: adolescents: 0.28 ± 0.13 mm, young adult: 0.28 ± 0.14 mm, *t*(34) <0.001, *p* = 1]. Moreover, the mean relative displacement and the absolute displacement were not correlated with age either in the migraine group (*Relative displacement r* = -0.04, *p* = 0.80; *Absolute displacement r* = -0.19, *p* = 0.26) or in the HC (*Relative displacement r* = 0.11, *p* = 0.51; *Absolute displacement r* = -0.12, *p* = 0.49).

### Group-Level ICA

**FIGURE 1:**
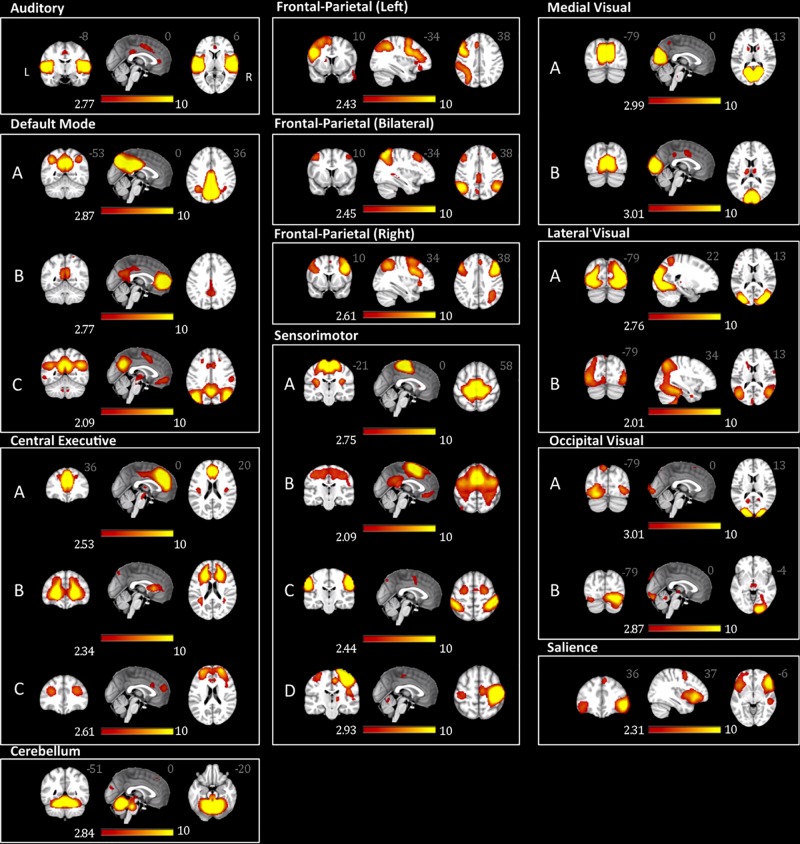
Adolescents and young adults resting-state networks. The networks were obtained using probabilistic ICA on all participants as implemented in MELODIC. The figure depicts the 22 components (from the 75 ICs of the group ICA) selected for subsequent analysis based on the Pearson spatial correlation with previously defined resting-state networks (Smith et al., 2009 19620724) and visual inspection. These 22 components corresponded to 10 distinct resting-state networks. The resting-state networks are represented as *z*-scores.

From the 75 ICs of the group ICA, 27 components were selected for the analysis based on Pearson spatial correlation with previously defined resting-state networks (Smith et al., 2009). From them, 5 components were discarded after visual inspection, remaining 22 components (Table 3). These 22 components corresponded to 10 different resting-state networks: auditory (AN), SN, DMN, medial visual (medial VN), lateral visual (lateral VN), occipital visual (occipital VN), SMN, CEN, FPN, and CER networks. Most of them were found in more than one component. For example, the FPN was split in three components: right, left, and bilateral FPN. The DMN was separated in three components one mostly posterior (DMN A) and two anterior-posterior (DMN B and DMN C). The CEN was divided in three components (CEN A, B, and C). The SMN was found on four components (SMN A, B, C, and D) with SMN D mostly lateralized on the right, and each VN was also found in two different components (A and B). The ICs and resting state networks are described in Figure 1 and Table 3.

**Table 3:** Components comprising resting-state network in relation to previously described resting-state network in Smith et al. (2009).

Smith template network	Independent component number (ICA)	Pearson’s r correlation
Medial VN -A	1	0.65
Medial VN -B	2	0.49
DMN -A	4	0.50
DMN -B	7	0.37
DMN -C	36	0.44
Lateral VN -A	8	0.55
Lateral VN -B	63	0.49
Auditory	9	0.62
SMN -A	10	0.31
SMN -B	22	0.39
SMN -C	24	0.27
SMN -D	37	0.28
CEN -A	11	0.40
CEN -B	17	0.29
CEN -C	54	0.31
FPN right	20	0.31
FPN left	16	0.48
FPN bilateral	26	0.31
Salience	39	0.41
Occipital VN -A	40	0.59
Occipital VN -B	67	0.34
Cerebellum	43	0.56


*VN, visual network; DMN, default-mode network; SMN, sensorimotor network; CEN, central executive network; FPN, frontal-parietal network, ICA, independent component analysis*.

### rs-FC Within Adolescent Groups (Migraine vs. HC)

**FIGURE 2:**
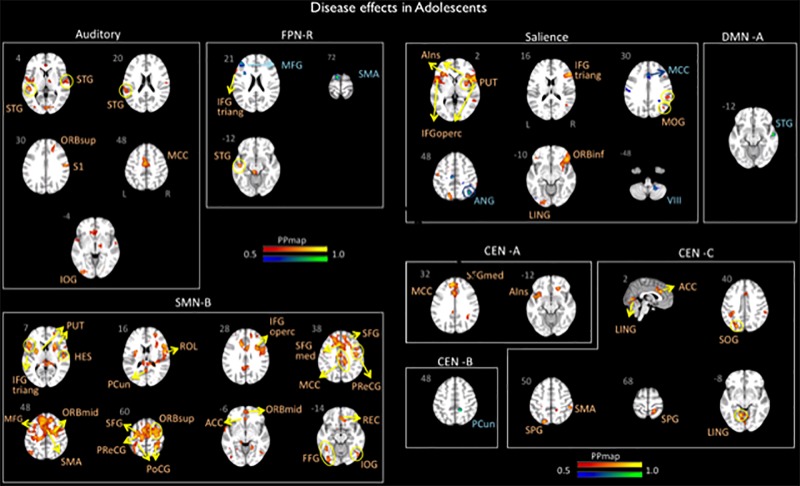
Disease effects on rs-FC networks in the adolescents group vs. healthy controls. The figure summarizes the statistically significant changes in connectivity measured between adolescent migraineurs and their healthy controls. Changes were found in the auditory, frontal parietal right, sensorimotor B, salience, default mode A, and central executive A, B, and C networks. See text for further details and Table 4. Numbers refer to the standard MNI Atlas coordinates. Abbreviations of brain regions are described in Table 8. Red – Yellow: Increased connectivity in adolescent migraineurs vs. controls. Blue – Green: Decreased connectivity in adolescent migraineurs vs. controls.

Significant differences in brain network connectivity between the adolescents with migraine and matched HC were found in the AN, CEN A, B and C, DMN A, right FPN, SN and SMN B (Table 4 and Figure 2).

**Table 4:** Disease effects in adolescents.

	Brain region	Lat	X (mm)	Y (mm)	Z (mm)	Vol (cm)	Z-stat
**Auditory**							
Migraine > Control	Cortical						
	*Frontal*						
	Sup. Orbital	R	18	34	32	1.24	2.92
	*Parietal*						
	Supramarginal	R	58	-14	28	1.8	4.22
	*Occipital*						
	Inferior	L	-34	-86	-4	1.03	3.12
	*Temporal*						
	Superior	R	62	-6	4	1	2.70
		L	-62	-34	20	2.55	2.87
	Middle	L	-50	-34	4	1.61	3.59
	Cingulum						
	Middle	L	-2	-6	48	2.79	3.94
**CEN –A (10)**							
Migraine > Control	Cortical						
	*Frontal*						
	Sup. Medial	L	-2	46	28	10.18	4.69
	Cingulum						
	Middle	L	2	18	36	1.97	3.78
		L	-6	-38	36	1.39	3.64
	Insula						
	Anterior	L	-42	6	-12	8	3.69
**CEN –C (53)**							
Migraine > Control	Cortical						
	*Parietal*						
	Supramarginal	R	54	-34	48	1.8	3.86
	Superior	R	18	-46	68	1.08	3.31
		L	-18	-74	52	1.42	3.75
	*Occipital*						
	Superior	L	-22	-74	40	1.25	3.77
	*Temporal*						
	Lingual	L	-10	-58	-8	1.46	4.18
		R	2	-70	0	1.49	4.04
	Cingulum						
	Anterior	L	2	26	28	2.06	4.33
**CEN –B (16)**							
Migraine < Control	Cortical						
	*Parietal*						
	Precuneus	R	14	-42	48	1.13	4.39
**DMN – A (03)**							
Migraine < Control	Cortical						
	*Temporal*						
	Superior	R	58	-6	-12	1.03	4.59
**FPN Right**							
Migraine > Control	Cortical						
	*Frontal*						
	Inferior triangular	L	-54	22	24	1.67	3.27
	*Temporal*						
	Superior	L	-46	-14	-12	3.41	4.17
Migraine < Control	Cortical						
	*Frontal*						
	Middle	L	-46	42	16	4.58	3.89
	SMA	L	-10	2	72	1.17	3.99
**Salience**							
Migraine > Control	Cortical						
	*Frontal*						
	Inf. Orbital	R	46	30	-8	4.18	3.34
	Inf. Triangular	R	54	26	16	1.65	3.36
	Inf. Operculum	L	-54	14	0	1.26	3.12
		R	58	10	4	5.09	3.76
	*Parietal*						
	Supramarginal	R	58	-38	36	1.02	2.53
	Superior	L	-14	-74	48	1.52	3.36
	*Occipital*						
	Middle	R	46	-66	24	1.04	3.13
	*Temporal*						
	Lingual	L	-18	-90	-12	1.15	2.99
	Insula						
	Anterior	L	-30	30	4	1.92	3.33
		R	34	22	4	2.09	2.91
	Sub-cortical						
	Putamen	R	30	6	4	2.1	2.67
Migraine < Control	Cortical						
	*Parietal*						
	Angular	R	42	-62	48	1.46	3.88
	Cingulum						
	Middle	L	-2	22	32	2.62	3.41
	Brainstem/cerebellum						
	Cerebellum 8	R	22	-54	-48	1.29	3.42
**SMN –B (21)**	Cortical						
Migraine > Control	*Frontal*						
	Mid. Orbital	L	-2	54	-4	1.47	3.19
		R	26	-2	48	9.7	4.05
		R	42	-2	56	1.44	2.79
	Inf. Orbital	L	-22	10	-24	1.05	3.64
	Sup. Orbital	R	30	2	60	2.06	3.27
		R	30	-14	60	1.73	3.78
	Inf. Triangular	L	-46	18	8	1.09	2.72
	Inf. Operculum	R	58	14	16	1.13	3.43
		R	34	6	28	2.28	3.07
	SMA	R	2	10	52	3.7	3.12
		R	6	18	56	2.7	3.08
		L	-10	2	60	3.07	2.99
		R	6	-14	64	3.8	2.67
		R	14	-18	68	1.26	2.55
	Precentral	R	46	6	40	1.94	2.7
		L	-30	-18	56	8.14	3.28
		R	46	-10	36	1.42	3.3
	Middle	L	-30	18	44	2.45	2.76
	Superior	L	-22	-2	56	1.1	2.53
		R	10	42	40	7.58	2.83
	Rectus	R	6	34	-16	1.14	2.88
	Sup. Medial	L	-2	22	40	14.74	3.35
	*Parietal*						
	Postcentral	R	34	-34	60	1.28	2.6
		L	-30	-38	64	3.85	2.81
		R	30	-46	56	2.45	3.74
	Precuneus	L	-2	-58	16	1.85	2.64
	*Occipital*						
	Rolandic Operculum	R	54	2	16	2.28	3.28
	Inferior	R	50	-70	-16	1.33	2.84
	*Temporal*						
	Superior	L	-62	-6	0	1.99	3.18
	Heschl	R	54	-14	8	1.93	3.16
	Fusiform	L	-42	-54	-12	1.04	3.13
		L	-34	-82	-16	1.74	2.69
	Cingulum						
	Anterior	R	2	34	-8	1.84	2.83
		R	6	10	28	1.56	2.88
	Middle	R	10	14	40	1.38	2.59
		R	2	2	44	1.36	2.5
		L	-6	-10	36	2.66	3.17
		R	10	-26	36	1.39	3.19
	Sub-cortical						
	Putamen	R	26	18	4	1.89	3.39
		L	-26	-2	8	2.19	3.31


*The table indicates brain areas of significant increased or decreased connectivity with the listed networks for adolescents with migraine vs. healthy controls. Coordinates and max statistical value (z-stat) are given for peak activity as well as volume (Vol) of each cluster of activity. “Lat” indicates the brain laterality (i.e., R, right side, L, left side)*.

#### Auditory

The AN displayed increased connectivity for adolescent migraineurs in comparison to controls. Increased connectivity was observed for the following cortical areas: frontal superior orbital, supramarginal parietal, inferior occipital, superior and middle temporal, and the middle cingulum.

#### Control Network

Increased and decreased connectivity were observed in the CEN in comparison to controls. In the CEN A, increased connectivity was observed cortically for the superior medial frontal, the middle cingulum and the anterior insula. Increased connectivity was also observed between the CEN C and the supramarginal and superior parietal, the temporal lingual, the superior occipital, and the anterior cingulum. Decreased connectivity in adolescent migraineurs in comparison to controls was only observed between the CEN B and the parietal precuneus.

#### Default Mode Network

The DMN A (posterior) displayed only a decreased connectivity in adolescent migraineurs with the superior temporal cortical area.

#### Right FPN

The right FPN displayed increased connectivity in adolescent migraineurs with the frontal inferior triangular and temporal superior areas. Decreased connectivity was also observed with the middle frontal and the supplementary motor area (SMA).

#### Salience Network

The SN displayed cortical and sub-cortical increased/decreased connectivity in the adolescents with migraine in comparison to matched HC. Increased connectivity was observed cortically with the inferior orbital, inferior triangular, and inferior operculum frontal, the supramarginal and superior parietal, the middle occipital, the lingual temporal and the anterior insula. Sub-cortically increased connectivity was observed with the putamen. Decreased connectivity in comparison to controls was also observed with the angular parietal, middle cingulum and cerebellar subdivision 8.

#### SMN

Increased connectivity was found between the SMN B and the following cortical and subcortical regions in adolescents with migraine in comparison to HC: inferior triangular, inferior operculum, SMA, precentral, middle, superior, rectus, superior medial, middle orbital, inferior orbital and superior orbital frontal, precuneus and postcentral parietal, inferior and rolandic operculum occipital, fusiform, Heschl, and superior temporal, anterior and middle cingulum, and the putamen.

### rs-FC Within Young Adult Groups (Migraine vs. HC)

**FIGURE 3:**
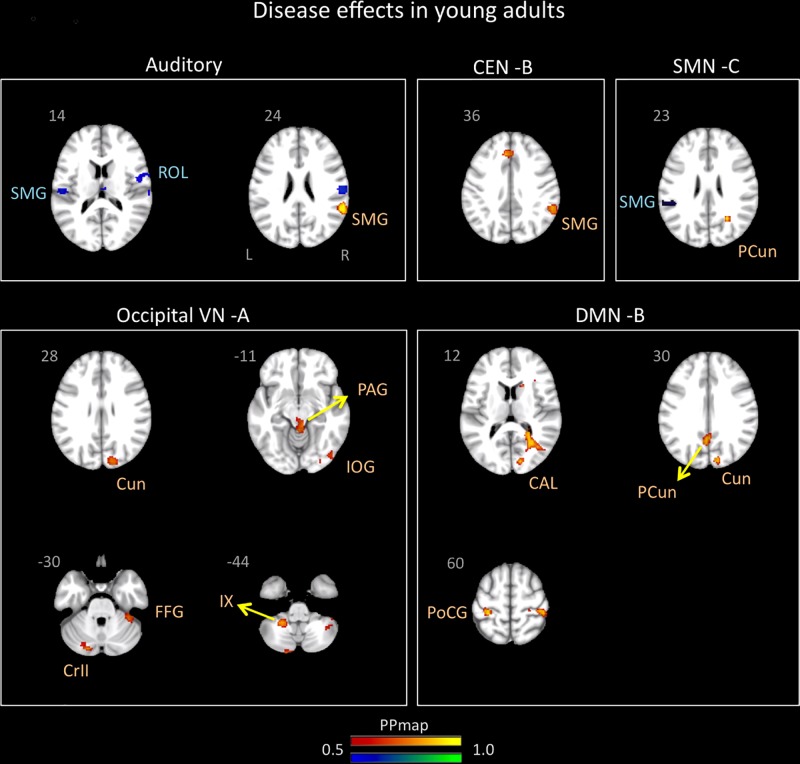
Disease effects on rs-FC networks in the young adults group vs. healthy controls. The figure summarizes the statistically significant changes in connectivity measured between young adult migraineurs and their healthy controls. Changes were found in the auditory, central executive B, sensorimotor C, occipital visual A, and default mode B networks. See text for further details and Table 5. Numbers refer to the standard MNI Atlas coordinates. Abbreviations of brain regions are described in Table 8. Red – Yellow: Increased connectivity in young adult migraineurs vs. controls. Blue – Green: Decreased connectivity in young adult migraineurs vs. controls.

Significant differences in brain network connectivity due to migraine in young adults in comparison to controls were found in the AN, CEN B, DMN B, SMN C, and occipital VN A (Table 5 and Figure 3).

**Table 5:** Disease effects in young adults.

	Brain region	Lat	X (mm)	Y (mm)	Z (mm)	Vol (cm)	Z-stat
**Auditory**							
Migraine > Control	Cortical						
	*Parietal*						
	Supramarginal	R	58	-46	24	1.51	4.51
Migraine < Control	Cortical						
	*Parietal*						
	Supramarginal	R	62	-18	24	1.43	3.27
		L	-54	-22	16	1.16	3.17
	*Occipital*						
	Rolandic Operculum	R	62	2	12	1.78	3.1
**DMN – B (6)**							
Migraine > Control	Cortical						
	*Parietal*						
	Postcentral	L	-30	-34	60	1.03	2.97
	Precuneus	L	-2	-62	36	2.49	3.21
	*Occipital*						
	Calcarine	R	30	-62	12	1.59	2.71
	Cuneus	R	14	-86	24	1.04	3.08
**CEN – B (16)**							
Migraine > Control	Cortical						
	*Parietal*						
	Supramarginal	R	58	-46	36	1.86	3.51
**SMN – C (23)**							
Migraine > Control	Cortical						
	*Parietal*						
	Precuneus	R	26	-58	28	1.11	4.88
Migraine < Control	Cortical						
	*Parietal*						
	Supramarginal	L	-50	-38	24	1.63	3.59
**Occipital VN – A (39)**							
Migraine > Control	Cortical						
	*Occipital*						
	Inferior	R	46	-74	-16	1	3.18
	Cuneus	R	14	-86	28	1.23	3.04
	*Temporal*						
	Fusiform	R	38	-38	-28	1.05	2.93
	Brainstem/Cerebellum						
	PAG		6	-30	-8	1.38	3.03
	Cerebellum 9	L	-22	-46	-44	1.08	3.74
	Cerebellum Crus 2	L	-18	-82	-32	1.37	3.69


*The table indicates brain areas of significant increased or decreased connectivity with the listed networks for young adults with migraine vs. healthy controls. Coordinates and max statistical value (z-stat) are given for peak activity as well as volume (Vol) of each cluster of activity. “Lat” indicates the brain laterality (i.e., R, right side, L, left side)*.

#### Auditory

The AN showed increased connectivity in young adults in comparison to controls with the supramarginal parietal and decreased connectivity with the supramarginal parietal and the rolandic operculum.

#### Default Mode Network

The DMN B (anterior/posterior) displayed only increased connectivity with the following cortical areas: postcentral and precuneus parietal, cuneus, and calcarine occipital.

#### Control Network

The CEN B displayed an increased in connectivity in young adults with migraine in comparison to HC in the supramarginal parietal area.

#### SMN

The SMN C displayed increased connectivity with the precuneus parietal and decreased connectivity with the supramarginal parietal.

#### Occipital VN A

The occipital VN showed only increased connectivity in different brain regions: inferior and cuneus occipital and fusiform temporal. This network was also the only one in young adults with migraine in comparison to controls to display increased connectivity in subcortical regions that included the PAG and the cerebellar subdivisions 9 and crus 2.

### rs-FC Between Adolescent and Young Adult Migraineurs Groups

**FIGURE 4:**
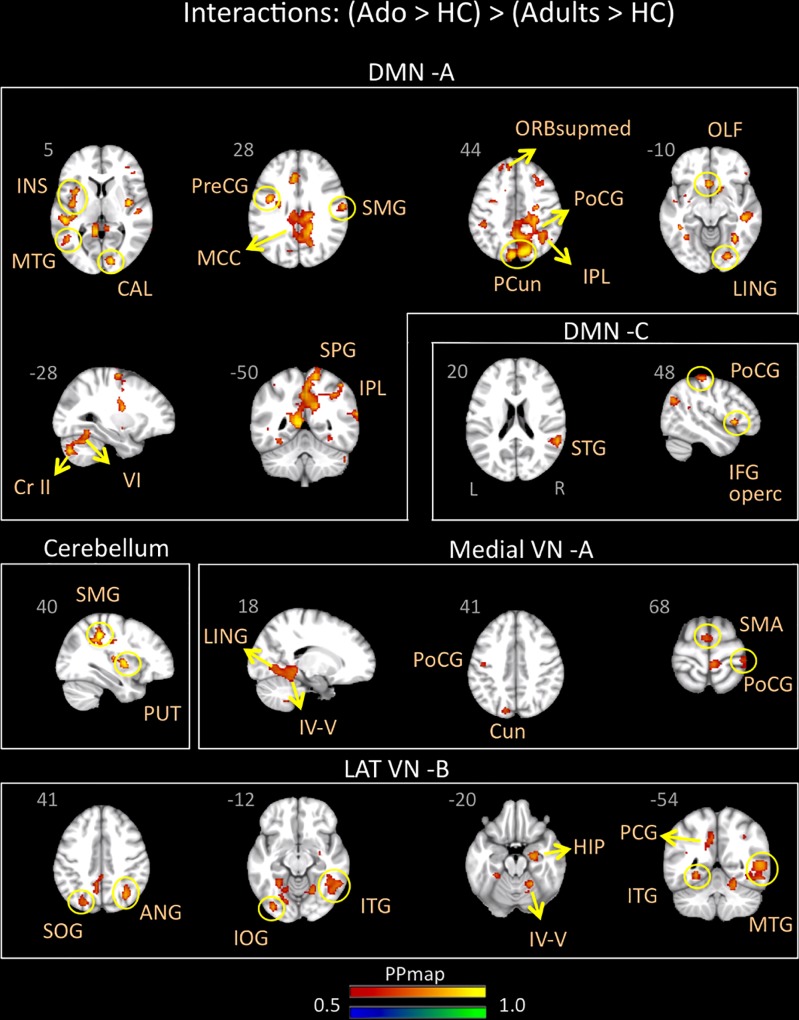
Interaction of disease effect by age [adolescents (Ado > HC) > young adults (Adults > HC)]. The figure summarizes the statistically significant greater rs-FC in adolescents with migraine in comparison to young adults with migraine and their healthy controls. Greater connectivity was found in the default-mode A and C, cerebellum, medial visual A, and lateral visual B networks. See text for further details and Table 6. Numbers refer to the standard MNI Atlas coordinates. Abbreviations of brain regions are described in Table 8. Red – Yellow: increased connectivity in adolescents migraineurs vs. young adults migraineurs. Blue – Green: Decreased connectivity in adolescents migraineurs vs. young adults migraineurs.

Adolescents with migraine exhibited greater connectivity in comparison to young adults with migraine and their HC (significant migraine-related age differences) in the CER, DMN A (posterior) and C (anterior/posterior), lateral VN B and medial VN A. In the CER, increased connectivity in comparison to the adult group was found with the parietal supramarginal region and the putamen; in the DMN A, was found with the following regions: medial superior, olfactory and precentral frontal, supramarginal, postcentral, superior, inferior and precuneus parietal, calcarine occipital, middle and lingual temporal, middle cingulum, anterior and posterior insula, cerebellar subdivision 6 and crus 2; and in the DMN C, with the inferior operculum, parietal postcentral, and superior temporal regions. In the lateral VN B, greater connectivity was observed with the following regions: parietal angular, inferior and superior occipital, middle, inferior and lingual temporal, posterior cingulum, hippocampus and cerebellar subdivision 4 and 5. Finally, the medial VN A displayed greater connectivity with the SMA, parietal postcentral, occipital cuneus, lingual temporal and cerebellar subdivision 4 and 5 (Table 6 and Figure 4).

**Table 6:** Interaction between groups – Adolescents (Mg > Hc) > Young adults (Mg > Hc).

	Brain region	Lat	X (mm)	Y (mm)	Z (mm)	Vol (cm)	Z-stat
Cerebellum							
	Cortical						
	*Parietal*						
	Supramarginal	R	34	-38	40	2.64	4.43
	Sub-cortical						
	Putamen	R	34	-2	4	1.26	3.78
DMN – A (03)	Cortical						
	*Frontal*						
	Medial superior	L	-6	42	48	1.11	2.55
	Olfactory	L	-2	18	-16	1.7	3.54
	Precentral	L	-38	-2	28	1.55	3.12
		L	-30	-10	64	1.31	3.06
	*Parietal*						
	Supramarginal	R	58	-14	24	1.71	3.3
	Postcentral	R	26	-38	48	1.49	3.34
	Precuneus	R	2	-46	52	2.30	2.97
		L	-6	-46	8	6.86	4.14
		R	6	-58	32	9.77	3.07
		R	18	-70	40	2.29	3.95
		R	14	-74	44	3.08	4.06
		L	-6	-82	44	3.33	4.05
	Superior	R	14	-50	64	1.39	3.01
	Inferior	R	46	-50	56	1.66	2.71
		R	38	-54	44	1.42	2.51
	*Occipital*						
	Calcarine	R	14	-86	4	2.55	3.44
	*Temporal*						
	Middle	L	-50	-30	0	2.47	3.93
		L	-46	-58	8	1.36	2.92
	Lingual	R	22	-82	-8	1.26	3.07
	Cingulum						
	Middle	L	-6	-42	36	1.43	2.41
	Insula						
	Anterior	L	-34	10	4	1.26	3.01
	Posterior	L	-34	-10	16	2.17	3.35
		L	-42	-10	4	1.18	2.82
	Cerebellum						
	Cerebellum 6	L	-22	-58	-28	1.8	3.64
	Cerebellum crus 2	L	-30	-78	-40	3.33	3.59
DMN – C (35)							
	Cortical						
	*Frontal*						
	Inferior Operculum	R	50	18	0	1.55	4.09
	*Parietal*						
	Postcentral	R	46	-26	60	1.07	3.26
	*Temporal*						
	Superior	R	58	-46	20	1.44	3.34
Lateral VN – B (62)							
	Cortical						
	*Parietal*						
	Angular	R	34	-66	44	2.02	3.95
		R	38	-66	48	1.27	3.82
	*Occipital*						
	Superior	L	-22	-78	40	1.44	2.84
	Inferior	L	-34	-82	-12	1.69	3.35
	*Temporal*						
	Middle	R	54	-54	4	2.93	3.53
	Inferior	R	50	-54	-12	1.33	2.85
	Lingual	L	-26	-54	-8	1.37	3.70
		L	-14	-82	-4	1.28	3.20
	Cingulum						
	Posterior	L	-10	-50	32	1.08	2.96
	Sub-cortical						
	Hippocampus	R	26	-14	-20	1	3.42
	Cerebellum						
	Cerebellum_4_5	R	22	-50	-20	1.06	3.67
Medial VN – A (00)							
	Cortical						
	*Frontal*						
	SMA	L	-2	-6	72	1.76	3.61
	*Parietal*						
	Postcentral	L	-46	-14	44	1.78	4.02
		R	38	-30	64	1.3	3.67
	*Occipital*						
	Cuneus	L	-14	-82	36	1.98	3.04
	*Temporal*						
	Lingual	R	26	-46	-4	1.44	3.13
		R	18	-62	-8	1.37	3.06
	Cerebellum						
	Cerebellum_4_5	R	18	-46	-16	1.35	3.39


*The table indicates brain areas of significant increased connectivity with the listed networks for adolescents with migraine vs. young adults with migraine and healthy controls. Coordinates and max statistical value (z-stat) are given for peak activity as well as volume (Vol) of each cluster of activity. “Lat” indicates the brain laterality (i.e., R, right side, L, left side)*.

**FIGURE 5:**
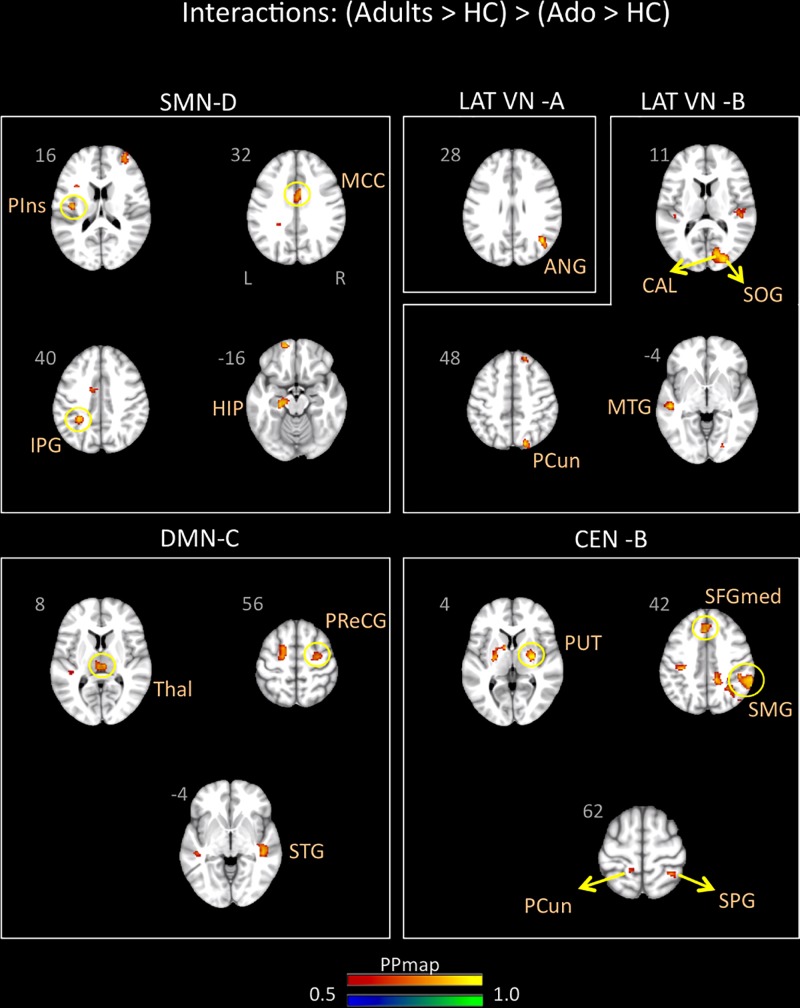
Interaction of disease effect by age [young adults (Adults > HC) > adolescents (Ado > HC)]. The figure summarizes the statistically significant greater rs-FC in young adults with migraine in comparison to adolescents with migraine and their healthy controls. Greater connectivity was found in the sensorimotor D, lateral visual A and B, default mode C, and central executive B networks. See text for further details and Table 7. Numbers refer to the standard MNI Atlas coordinates. Abbreviations of brain regions are described in Table 8. Red – Yellow: Increased connectivity in adult migraineurs vs. adolescent migraineurs and their respective healthy controls. Blue – Green: Decreased connectivity in adult migraineurs vs. adolescent migraineurs and their respective healthy controls.

Young adults with migraine exhibited greater connectivity in comparison to adolescents with migraine and their HC (significant migraine-related age differences) in the SMN D (lateralized on the right), DMN C, CEN B, and lateral VN A and B. In the SMN D, greater connectivity was found with the inferior parietal, the middle cingulum, the posterior insula, and the hippocampus. In the DMN C, greater connectivity was found with the frontal precentral area, the superior temporal region and the thalamus. In the CEN B, greater connectivity was found with the frontal superior medial, the supramarginal, precuneus and superior parietal, and the putamen. Finally, greater connectivity was also found between the lateral VN A and the angular cortex, and the lateral VN B and the precuneus parietal, the superior and calcarine occipital, and the middle temporal cortex (Table 7 and Figure 5).

**Table 7:** Interaction between groups – Young adults (Mg > Hc) > Adolescents (Mg > Hc).

	Brain region	Lat	X (mm)	Y (mm)	Z (mm)	Vol (cm)	Z-stat
Cerebellum							
	Brain region	Lat	X (mm)	Y (mm)	Z (mm)	Vol (cm)	Z-stat
DMN – C (35)							
	Cortical						
	* Frontal*						
	Precentral	R	30	-10	56	1	3.09
	*Temporal*						
	Superior	R	46	-26	-4	1.3	3.69
	Parahippocampus						
	Thalamus	R	6	-22	8	1.61	3.42
CEN – B (16)							
	Cortical						
	*Frontal*						
	Superior medial	L	-2	26	48	1.03	3.53
		L	2	34	40	1.26	3.33
	*Parietal*						
	Supramarginal	R	62	-38	36	7.06	4.53
	Precuneus	L	-14	-42	68	1.01	3.54
	Superior	R	34	-46	60	1.16	3.62
	Parahippocampus						
	Putamen	R	30	-10	4	1.15	4.06
SMN – D (36)							
	Cortical						
	*Parietal*						
	Inferior	L	-30	-42	40	1.14	3.18
	Cingulum						
	Middle	R	2	-2	32	1.38	2.63
	Insula						
	Posterior	L	-38	-14	16	1.28	3.12
	Sub-cortical						
	Hippocampus	L	-22	-22	-16	1.29	3.29
							
Lateral VN – A (07)							
	Cortical						
	*Parietal*						
	Angular	R	42	-62	28	1.02	3.89
Lateral VN – B (62)							
	Cortical						
	*Parietal*						
	Precuneus	R	18	-78	48	1.92	3.43
	*Occipital*						
	Calcarine	R	18	-78	8	1.65	3.51
	Superior	R	26	-86	12	1.27	3.45
	*Temporal*						
	Middle	L	-50	-26	-4	1.23	3.55
							


*The table indicates brain areas of significant increased connectivity with the listed networks for young adults with migraine vs. adolescents with migraine and healthy controls. Coordinates and max statistical value (z-stat) are given for peak activity as well as volume (Vol) of each cluster of activity. “Lat” indicates the brain laterality (i.e., R, right side, L, left side)*.

**Table 8:** Abbreviations of brain regions.

Brain regions	Abbr.	Brain regions	Abbr.
Anterior insula	AINS	Postcentral gyrus	PoCG
Anterior cingulate cortex	ACC	Posterior cingulate gyrus	PCG
Angular gyrus	ANG	Precentral gyrus	PreCG
Calcarine fissure	CAL	Precuneus	PCUN
Cuneus	CUN	Posterior insula	PINS
Fusiform gyrus	FFG	Putamen	PUT
Gyrus rectus	REC	Rolandic operculum	ROL
Heschl gyrus	HES	Superior frontal gyrus	SFG
Hippocampus	HIP	Superior frontal gyrus, medial	SFGmed
Inferior frontal gyrus, opercular part	IFGoperc	Superior frontal gyrus, orbital part	ORBsup
Inferior frontal gyrus, orbital part	ORBinf	Superior frontal gyrus, medial orbital	ORBsupmed
Inferior frontal gyrus, triangular part	IFGtriang	Superior Occipital gyrus	SOG
Inferior occipital gyrus	IOG	Superior parietal gyrus	SPG
Inferior temporal gyrus	ITG	Superior temporal gyrus	STG
Insula	INS	Superior temporal pole	STmP
Lingual gyrus	LING	Supplementary motor cortex	SMA
Middle frontal gyrus	MFG	Supramarginal gyrus	SMG
Middle frontal gyrus, orbital part	ORBmid	Thalamus	Thal
Middle cingulate cortex	MCC	Cerebellum Crus II	CRII
Middle occipital gyrus	MOG	Cerebellar lobule IV–V	IV–V
Middle temporal gyrus	MTG	Cerebellar lobule VIII	VIII
Olfactory cortex	OLF	Cerebellar lobule IX	IX
Periaqueductal gray gyrus	PAG		


**FIGURE 6:**
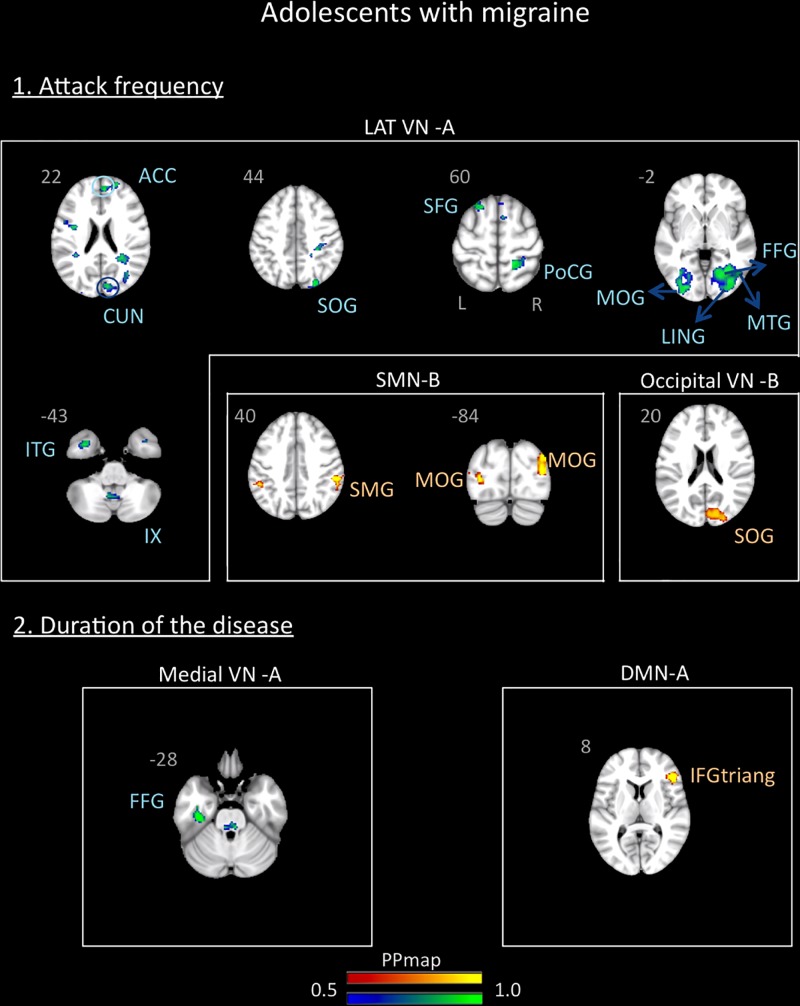
Association between the rs-FC of the adolescents with migraine and two clinical indicators of the disease: attack frequency (per month) and duration of the disease (in years). The figure shows significant positive association between attack frequency and the sensorimotor B and occipital visual B networks and negative association with the lateral visual A network. The duration of the disease was positively associated with the default mode network A and negatively associated with the medial visual A network. See text for further details and Supplementary Table 1. Numbers refer to the standard MNI Atlas coordinates. Abbreviations of brain regions are described in Table 8.

**FIGURE 7:**
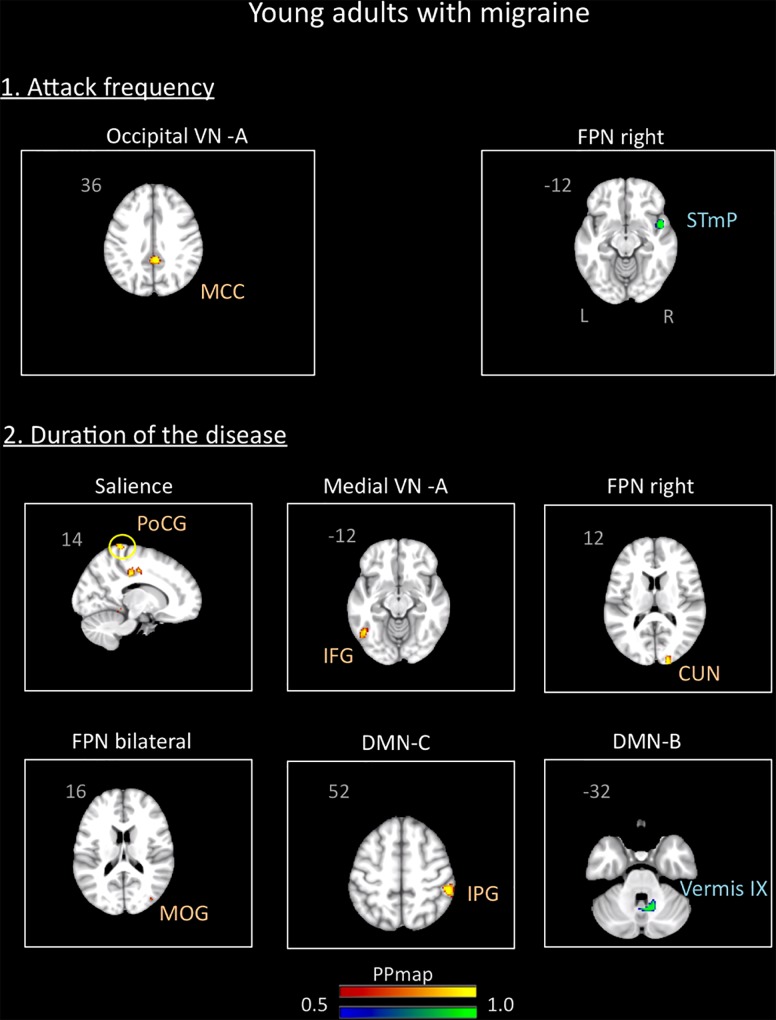
Association between the rs-FC of the young adults with migraine and two clinical indicators of the disease: attack frequency (per month) and duration of the disease (in years). The figure shows significant positive association between attack frequency and the occipital visual A network and negative association with the right frontal parietal network. The duration of the disease was positively associated with the salience, medial visual A, right frontal parietal, bilateral frontal parietal, and default mode C networks and negatively associated with the default mode network B. See text for further details and Supplementary Table 2. Numbers refer to the standard MNI Atlas coordinates. Abbreviations of brain regions are described in Table 8.

*The table indicates the abbreviations used in Figure 2–7 to describe brain regions*.

### rs-FC and Disease Manifestations (Duration and Frequency) Within Adolescent and Young Adult Groups

In the adolescent group, the correlation analysis revealed no significant association between the variables “duration of the disease (in years)” and “attack frequency (per months)” (Pearson *r* = 0.13, *p* = 0.59). There was also no correlation between age and these variables (age and attack frequency: *r* = -0.19, *p* = 0.45; age and duration: *r* = 0.33, *p* = 0.18). The functional connectivity data revealed association with the frequency of the attacks and a small association with the duration of the disease. The frequency of the attacks was positively associated with the SMN B and the Occipital VN B and negatively associated with the lateral VN A. The duration of the disease was positively associated with the DMN A and negatively associated with the medial VN A. Details are described in Supplementary Table 1 and Figure 6.

In the young adults group, the correlation analysis revealed no significant association between the variables “duration of the disease (in years)” and “attack frequency (per month)” (Pearson *r* = -0.19, *p* = 0.94). There was also no correlation between age and these variables (age and attack frequency: *r* = 0.18, *p* = 0.46, *p* = 0.4; age and duration: *r* = -0.9, *p* = 0.72). The results revealed association of the functional connectivity with disease duration and attack frequency. Small positive associations were found for attack frequency with the occipital VN A and small negative associations with the right FPN. Duration of the disease was positively associated with the medial VN A, the right and bilateral FPN, the SN, and the DMN C. Negative association was also found with the DMN B. Details are described in Supplementary Table 2 and Figure 7.

## Discussion

Here we investigated differences in whole brain rs-FC within and between adolescents and young adults with migraine and a group of age- and sex-matched healthy controls. Our RSN’s showed similar patterns for those previously reported in the literature (Table 3 and Figure 1). The significant findings can be summarized as follows: (1) in the within-group comparisons, more widespread abnormal functional connectivity in the adolescents than in the young adults and more abnormal functional connectivity associated with sensory networks in the young adults; (2) in the between-group comparisons, more rs-FC changes in the DMN and stronger rs-FC in the cerebellum network in the adolescents than in the young adults, and stronger rs-FC in the CEN and SMN in the young adults; and (3) differences in the two groups related to rs-FC and disease manifestations (duration and frequency). With respect to migraine, network differences underlie clinical and subclinical behavioral changes related to the disease presentation, for example, reward processing (Wilcox et al., 2016), cognition (Mathur et al., 2015), sensation (Moulton et al., 2011; Stankewitz and May, 2011), or differences in disease load (Borsook et al., 2012), or disability (Steiner et al., 2016). Such changes in brain function in migraineurs may be different with later childhood (with brain development) vs. early adulthood. As noted by others, significant differences in neuropsychological features are present in that young migraine brain (Oelkers-Ax et al., 2004, 2005).

### rs-FC Within Adolescent and Young Adult Groups (Migraine vs. HC)

In adolescents, the major effects of the disease relative to HC were noted in 6 networks – AN, CEN (A, B and C), SN, SMN B, FPN right, and DMN A with most changes showing increased rs-FC for the migraine group vs. HC (exception being the DMN A, the CEN B, some activations with the FPN right and the SN) (see Table 4 and Figure 2). As we expected, most of these networks are involved in higher cognitive functions processes and regulation of emotions, i.e., CEN, SN, FPN, and the DMN. These results could be linked to the maturation of cognitive, emotional and social processes that are still happening during adolescence (Paus et al., 2008). Although few data are available, our results are globally in accordance with previous studies investigating changes in seed-based rs-FC in pediatric populations suffering from chronic pain. One study from our group has reported significant abnormal functional connectivity in brain regions associated with sensory, motor and affective functions in pediatric females with migraine compared with males with migraine and HC (adolescents from 10 to 16 years) and more specifically greater rs-FC in migraineurs between (1) the precuneus and the putamen, caudate, thalamus, and amygdala, and between (2) the amygdala and thalamus, SMA, and anterior MCC (Faria et al., 2015). Notably, the precuneus is a central node of the DMN (Fransson and Marrelec, 2008) and the amygdala and part of the thalamus are reported to be part of the SN (Seeley et al., 2007). In adolescents with abdominal pain, seed-based analyses showed altered rs-FC within key nodes of the DMN and the cognitive control network (Hubbard et al., 2016). Moreover, changes in rs-FC in networks involved in high order cognitive functions (FPN, SN, DMN, CEN) as well as in the SMN and the cerebellum have been also observed in CRPS pediatric patients (Becerra et al., 2014).

Of the specific brain regions showing disease effects within networks, the temporal region is involved in almost all the networks showing significant changes, mainly increased rs-FC. We, and others, have previously noted alterations in the temporal brain region in adult migraineurs (Moulton et al., 2011; Coppola et al., 2015; Schwedt et al., 2015). The region is involved in diverse neural processing including but not limited to sound (Binder et al., 2000), smell (Jones-Gotman and Zatorre, 1993), visual (Baizer et al., 1991), and social and emotion (Olson et al., 2007). These rs-FC changes with temporal region could be linked to alteration of functional sensory processing and hypersensitivity to painful stimuli that have been suggested in adult migraine patients with potential persistence with less magnitude in the interictal period (Main et al., 1997; Moulton et al., 2011; Schwedt et al., 2011). Indeed, in the present study, adolescents reported accompanied sensory symptoms during their migraine attacks (photophobia, phonophobia, nausea, vomit). Moreover, increased rs-FC in AN and SMN networks were also found in the adolescents with migraine vs. HC as well as increased rs-FC between the anterior insula and the SN and CEN-A, and the anterior cingulate cortex (ACC) and the CEN-C and SMN-B. The insula and ACC are well known to be involved in pain processing (Peyron et al., 2000; Apkarian et al., 2005) and dysfunction of the SMN in adults migraineurs has been recently linked to potential disruption of nociceptive pathways (Zhang et al., 2017). Although speculative because sensitivity to painful stimuli has not been assessed in the current study, these rs-FC changes in adolescents could be also linked to a disruption of nociceptive pathways as observed in adults with migraine (Colombo et al., 2015; Schulte and May, 2016).

In young adults, differences (migraineurs > controls) were also noted in a great number of networks including the AN, CEN B, SMN C, DMN B and Occipital VN A (see Table 5 and Figure 3). The majority of these network differences involved sensory networks (SMN, AN, VN) with most changes showing increased rs-FC for the migraine group vs. HC (exception being for some activations with the AN and SMN C). Rs-FC changes with most of these networks have been demonstrated in previous studies with adult migraineurs, i.e., DMN (Xue et al., 2012); CEN (Xue et al., 2012); VN (Tedeschi et al., 2016); SMN (Zhang et al., 2017). Within these networks brain regions showing differences include cerebellar (including Crus 2) changes, a region involved in both sensory and affective processing in pain (Saab and Willis, 2003; Ruscheweyh et al., 2014); parietal precuneus, a region involved in a large spectrum of highly integrated tasks, in visuospatial integration and, a central node of the DMN (Fransson and Marrelec, 2008); and the periaqueductal gray (PAG), a region involved in a number of processes including pain modulation (Mainero et al., 2011; Chen et al., 2017). Alterations of PAG network connectivity with nociceptive and sensory processing pathways have also been noted in adult migraineurs (Mainero et al., 2011; Chen et al., 2017) and have been linked to an impairment of the descending pain modulatory circuits (Mainero et al., 2011). Of note, changes in rs-FC between the SN and cerebellar region, and between the SMN and the precuneus were also found in the adolescents vs. HC comparison. Finally, the increased presence of sensory related rs-FC may relate to migraine related processes involving conscious or non-conscious changes in pain, visual (photophobia), and auditory (phonophobia) driven by central sensitization (Burstein et al., 2010; Hodkinson et al., 2016). Globally, our results in young adults are in line with prior reports in adults showing that migraine is a process that affects the brain in a profound manner over time, including rs-FC changes in various brain regions and networks (e.g., pain processing network, affective network, DMN, CEN, SN, and VN) (Colombo et al., 2015; Schulte and May, 2016).

Noteworthy, we found partial overlap between alterations in rs-FC in adolescents vs. HC and young adults vs. HC, suggesting partly similar brain intrinsic connectivity changes in these two developmental time points. However, whether these common rs-FC alterations can be interpreted as a consequence of the repetition of the attacks or a condition that precedes the migraine disease remains unclear. Parts of these alterations could be the consequence of repeated attacks as we found more effect of the duration of the disease in young adults than in adolescents, but it is also possible that alterations in some networks would be the consequence of the migraine attacks and in other networks precede the disease (see Discussion). Moreover, more widespread rs-FC changes within and between networks were globally observed in the adolescents with migraine vs. HC than in the young adults with migraine vs. HC, as well as for the adolescents in the between group comparison (see section “Materials and Methods”). Although speculative, these results could be partly linked to the changes in connectivity strengths and network relationships still happening during adolescence (Stevens, 2016). Indeed, although the basic organization of FC networks is established by the age of 12 or even earlier depending on functional networks (Marek et al., 2015; Thornburgh et al., 2017), rs-FC changes continue during adolescence (Marek et al., 2015; Stevens, 2016). Major differences include the relative development of subcortical limbic systems vs. top-down control systems during adolescence compared to young adults as well as a shift from local to distal connectivity profiles and changes in specific connectivity strength among brain regions (Power et al., 2010; Wang et al., 2012; Baker et al., 2015; Ernst et al., 2015; Stevens, 2016). Typically, intrinsic brain connectivity of adolescents is at an intermediate rs-FC pattern, sharing aspects of both children and adults (Kelly et al., 2009) with potentially distinct developmental trajectories according to brain networks (Gu et al., 2015; Stevens, 2016).

### rs-FC Between Adolescent and Young Adult Migraineurs Groups

For the comparison [adolescents (Ado > HC) > young adults (Adults > HC)], we observed stronger rs-FC in adolescents with the CER, DMN A and C and lateral and medial VNs (see Table 6 and Figure 4). These results contrast with our expectation to find mostly stronger rs-FC in adolescents within networks involved in higher cognitive functions and emotion regulations (Paus et al., 2008). Nevertheless, stronger rs-FC in the DMN was prominent and included frontal (e.g., insula), parietal (e.g., precuneus, supramarginal), occipital (e.g., calcarine), temporal and cerebellar regions. Furthermore, whereas changes between rs-FC networks and cerebellar regions are shown in adolescents vs. HC and young adults vs. HC, we found only significant stronger rs-FC with the cerebellum network for this comparison. As mentioned above, there is evidence for the involvement of cerebellum in pain pathogenesis (Saab and Willis, 2003; Ruscheweyh et al., 2014) but also in a number of integrative cognitive, behavioral and sensory-motor functions (Saab and Willis, 2003). Increased rs-FC between the right cerebellum and the right medial prefrontal cortex has been noted in adults with migraine (Jin et al., 2013). Finally, as in the adolescents vs. HC comparison, the temporal region was also involved in almost all the networks showing significant rs-FC changes.

For the comparison [young adults (Adults > HC) > adolescents (Ado < HC)], we observed stronger rs-FC in young adults with the CEN, DMN, SMN, and lateral VN A and B (see Table 7 and Figure 5). Within these networks – brain region connectivity, regions showing increased connectivity in adults included sensory processing regions (posterior insula) with the SMN, and the posterior thalamus/pulvinar the region is involved in central sensitization (Burstein et al., 2010) with the DMN. Interestingly, the hippocampus, associated with stress and memory is also present in association with the SMN (Maleki et al., 2013).

The two comparisons showed the following overall differences: (1) There was a greater number of regions that showed stronger functional connectivity for [adolescents (Ado > HC) > young adults (Adults > HC)] for the DMN and the visual network; and (2) some networks showed stronger connectivity in only one group of age viz., cerebellum network for [adolescents (Ado > HC) > young adults (Adults > HC)] and CEN and SMN for [young adults (Ado > HC) > adolescents (Adults > HC)]. Alterations in FC within the DMN is associated with a number of diseases and disorders in adults (Gorges et al., 2013; Weiler et al., 2014) and in adolescents (Dalwani et al., 2014; Borich et al., 2015; Ho et al., 2015) and represents an approach to evaluating differences in brain function associated with a major network. Thus, altered DMN connectivity may be a target to evaluate differences in the evolution of the disease over time, particularly at vulnerable time-points of the disease (viz., puberty, menopause, etc.). In support of this notion, prior studies on alterations of the DMN in migraineurs have suggested in brain regions comprising the network or those with which the network interacts the following: (1) disruption of the DMN in adult migraineurs (Tessitore et al., 2013); (2) the role of the DMN and regions such as the insula may encode migraine headache (Coppola et al., 2018); (3) spontaneous migraine attacks alter DMN connectivity (Edes et al., 2017); (4) DMN may be a potential predictor of neuromodulation of migraine (Kinfe, 2018); and (5) increased DMN connectivity (Zhang et al., 2017), to mention a few. Taking the DMN as an example, but the theme applies to the differences found for the other RSN’s, the significant differences observed between the groups is an indicator of the dynamic state of brain processing with migraine “maturation.” In the present study, this theme applies to the other networks showing differences between the two groups.

### rs-FC and Disease Manifestations (Duration and Frequency) Within Adolescent and Young Adult Groups

Within each development group, we have assessed the relationship between rs-FC patterns and individual migraine characteristics (i.e., duration of migraine in years and attack frequency per month).

In the two groups, positive and negative associations were noted with baseline brain intrinsic connectivity. In adolescents, a few more associations were found between rs-FC patterns and attack frequency than with the duration of the disease (see Supplementary Table 1 and Figure 6). In the young adult group more rs-FC patterns were associated with the duration of the disease relative to the adolescent group and only a few associations were found between rs-FC patterns and attack frequency as well (see Supplementary Table 2 and Figure 7). As shown in Table 2, the duration of the disease was significantly higher in young adults with migraine than in adolescents and may explain the presence of more rs-FC changes associated to the duration of the disease. In previous studies, positive or negative associations between functional and structural brain abnormalities and disease duration and/or frequency of the disease have been also reported in adults with migraine (Yu et al., 2012; Jin et al., 2013; Xue et al., 2013; Hodkinson et al., 2015) and in some pediatric cohorts (Rocca et al., 2014; Youssef et al., 2017). These data suggest that part of the rs-FC brain changes found here in young adults may be linked to the migraine disease progression. Moreover, the fact that in adolescents only minor associations were found with migraine duration may suggest that part of the rs-FC network changes may arise before the onset of migraine in the adolescent group. In accordance with this hypothesis, some functional and structural brain studies in pediatric cohorts did not report any correlation between their results and the disease duration (Youssef et al., 2017; Messina et al., 2018).

### Caveats

The present study has a number of caveats including: (1) *migraine subgroups*: patients with episodic migraine within 48 and 72 h after the scan session were included; both migraine with or without aura were included; (2) *medication*: medication, including years of treatment were probably different according to the age of the patients; (3) *sex distribution*: although equal numbers of males and females were included, sex differences were not evaluated because we did not have sufficient numbers; it is possible, given prior reports of differences in brain structure and function in men and women (Maleki et al., 2013; Borsook et al., 2014; Faria et al., 2015) that the changes may be driven by sex as well as age; (4) *division of groups by age*: a further limitation is the somewhat arbitrary division of the sample by age. We have followed binning used in previous papers investigating developmental effect on functional networks (Williams et al., 2006; Kelly et al., 2009; Hwang et al., 2013; Rubia, 2013; Marek et al., 2015). Nevertheless, given prior reports of the potential interaction between onset of migraine and puberty (Borsook et al., 2014), binning based on a clinical measure of pubertal status or determination of sex hormones would be useful in future studies; and (5) *Movement*: It should be noted that while we have attempted our best to control for movement with the current procedures the field hasn’t settled on the optimal procedures. In the approach used here is it doesn’t get rid of the noise related to movement and respirations that are not spatially organized (e.g., that are across the whole brain) and thus future work in this domain is warranted.

## Conclusion

Our findings show differences rs-FC in migraineurs in pre and early adults when brain development is still ongoing and at a time when migraine prevalence is near its peak (Bigal and Lipton, 2009). The normal development as assessed by rs-FC is disrupted or different in migraineurs vs. healthy controls and within the migraine groups with development. Such changes provide a basis to further understand the neurobiology of the disease and formulation of different strategies for disease prediction and therapeutic opportunities.

## Author Contributions

DB, LB, and RB conceived and designed the study. EC, LB, SW, and AY analyzed the data. EC, LB, DB, DF, and AL prepared the manuscript. AAL and AL recruited the patients and collected the data. All authors edited the manuscript.

## Conflict of Interest Statement

The authors declare that the research was conducted in the absence of any commercial or financial relationships that could be construed as a potential conflict of interest.

## Footnotes

1 https://www.ichd-3.org

2 http://fsl.fmrib.ox.ac.uk/fsl

3 http://fsl.fmrib.ox.ac.uk/fsl/fslwiki/MELODIC

4 https://fsl.fmrib.ox.ac.uk/fsl/fslwiki/DualRegression

5 https://fsl.fmrib.ox.ac.uk/fsl/fslwiki/GLM

6 www.fmrib.ox.ac.uk/datasets/techrep
