# Supplementary material for: Migraine in the Young Brain: Adolescents vs. Young Adults
Source: Front Hum Neurosci. 2019 Mar 22;13:87. doi: 10.3389/fnhum.2019.00087 (PMC6438928; doi:10.3389/fnhum.2019.00087)
Supplement: Supplementary file 1 [file Table_1.docx]

Supplementary Material

**Migraine in the Young Brain: Adolescents vs. Young Adults**

Elisabeth Colon*, Allison Ludwick, Sophie L. Wilcox, Andrew M. Youssef, Amy Danehy, Damien A Fair, Alyssa Lebel_,_ Rami Burstein, Lino Beccera, and David Borsook.

Corresponding Author:

David Borsook, MD, PhD

c/o Center for Pain and the Brain

Boston Children’s Hospital and Harvard Medical School

1 Autumn Street, Boston MA 02115

Telephone number: (+1) 781-216-1199

Fax number: (+1) 781-216-1183

Email: [david.borsook@childrens.harvard.edu](mailto:david.borsook@childrens.harvard.edu)

**Supplementary Table 1:** Adolescents with migraine: Effect of attack frequency (month) and duration (years).

The table indicates brain areas of significant positive or negative association with the listed networks and “attack frequency” or “duration of the disease” in the adolescents with migraine. Coordinates and max statistical value (z-stat) are given for peak activity as well as volume (Vol) of each cluster of activity. “Lat” indicates the brain laterality (i.e., R = right side, L= left side).

| 1. Attack frequency (/month) | | | | | | | |
| --- | --- | --- | --- | --- | --- | --- | --- |
|  | Brain region | Lat | X (mm) | Y (mm) | Z (mm) | Vol (cm) | Z-stat |
| **SMN –B (21)** |  |  |  |  |  |  |  |
| Positive association | Cortical |  |  |  |  |  |  |
|  | *Parietal* |  |  |  |  |  |  |
|  | Supramarginal | R | 58 | -34 | 40 | 1.16 | 4.43 |
|  | *Occipital* |  |  |  |  |  |  |
|  | Middle | R | 42 | -82 | 16 | 1.41 | 4.07 |
|  |  | L | -34 | -86 | 4 | 1.06 | 3.8 |
| **Occipital VN–B (66)** |  |  |  |  |  |  |  |
| Positive association | Cortical |  |  |  |  |  |  |
|  | *Occipital* |  |  |  |  |  |  |
|  | Superior | R | 18 | -86 | 20 | 2.62 | 2.37 |
| **Lateral VN–A (07)** |  |  |  |  |  |  |  |
| Negative association | Cortical |  |  |  |  |  |  |
|  | *Frontal* |  |  |  |  |  |  |
|  | Superior | L | -22 | 22 | 60 | 1.04 | 3.28 |
|  | *Parietal* |  |  |  |  |  |  |
|  | Postcentral | R | 22 | -46 | 60 | 1.9 | 3.16 |
|  | *Temporal* |  |  |  |  |  |  |
|  | Fusiform | R | 30 | -66 | -4 | 7.24 | 3.31 |
|  | Inferior | L | -38 | -2 | -40 | 1.17 | 3.23 |
|  | Middle | R | 46 | -66 | 0 | 1.26 | 2.36 |
|  |  | R | 46 | -74 | 12 | 3.79 | 4.18 |
|  |  | R | 26 | -86 | -8 | 2.86 | 3.21 |
|  | Lingual |  |  |  |  |  |  |
|  | *Occipital* |  |  |  |  |  |  |
|  | Superior | R | 26 | -82 | 44 | 3.2 | 3.11 |
|  | Cuneus | R | 10 | -86 | 24 | 1.62 | 2.89 |
|  | Middle | L | -30 | -90 | 0 | 9.1 | 3.59 |
|  | *Cingulum* |  |  |  |  |  |  |
|  | Anterior | R | 6 | 50 | 20 | 1.13 | 2.94 |
|  |  |  |  |  |  |  |  |
|  | *Cerebellum* |  |  |  |  |  |  |
|  | Cerebellum 9 | L | -2 | -58 | -48 | 1.11 | 2.93 |
|  |  |  |  |  |  |  |  |
| 2. Duration of the disease (years) | | | | | | | |
|  | Brain region | Lat | X (mm) | Y (mm) | Z (mm) | Vol (cm) | Z-stat |
| **DMN –A (03)** |  |  |  |  |  |  |  |
| Positive association | Cortical |  |  |  |  |  |  |
|  | *Frontal* |  |  |  |  |  |  |
|  | Inferior Triangular | R | 46 | 30 | 8 | 1.38 | 5.35 |
| **Medial VN –A (00)** |  |  |  |  |  |  |  |
| Negative association | Cortical |  |  |  |  |  |  |
|  | *Temporal* |  |  |  |  |  |  |
|  | Fusiform | L | -34 | -22 | -28 | 1.34 | 3.76 |
|  |  |  |  |  |  |  |  |
|  |  |  |  |  |  |  |  |

**Supplementery Table 2**: Young adults with migraine: Effect of attack frequency (month) and duration (years).

The table indicates brain areas of significant positive or negative association with the listed networks and “attack frequency” or “duration of the disease” in the young adults with migraine. Coordinates and max statistical value (z-stat) are given for peak activity as well as volume (Vol) of each cluster of activity. “Lat” indicates the brain laterality (i.e., R = right side, L= left side).

| 1. Attack frequency (/month) | | | | | | | |
| --- | --- | --- | --- | --- | --- | --- | --- |
|  | Brain region | Lat | X (mm) | Y (mm) | Z (mm) | Vol (cm) | Z-stat |
| **FPN right (19)** | Cortical |  |  |  |  |  |  |
| Negative association | *Temporal* |  |  |  |  |  |  |
|  | Superior pole | R | 50 | 6 | -12 | 1 | 7.62 |
| **Occipital VN –A (39)** | Cingulum |  |  |  |  |  |  |
| Positive association | Middle | R | 2 | -42 | 36 | 2.23 | 4.89 |
|  |  |  |  |  |  |  |  |
|  |  |  |  |  |  |  |  |
|  |  |  |  |  |  |  |  |
| 2. Duration of the disease (years) | | | | | | | |
|  | Brain region | Lat | X (mm) | Y (mm) | Z (mm) | Vol (cm) | Z-stat |
| **Medial VN –A (00)** |  |  |  |  |  |  |  |
| Positive association | Cortical |  |  |  |  |  |  |
|  | *Temporal* |  |  |  |  |  |  |
|  | Inferior | L | -46 | -58 | -12 | 1.21 | 3.95 |
| **FPN right (19)** |  |  |  |  |  |  |  |
| Positive association | Cortical |  |  |  |  |  |  |
|  | *Occipital* |  |  |  |  |  |  |
|  | Cuneus | R | 18 | -98 | 12 | 1.04 | 4.91 |
| **FPN Bilateral** |  |  |  |  |  |  |  |
| Positive association | Cortical |  |  |  |  |  |  |
|  | *Occipital* |  |  |  |  |  |  |
|  | Middle | R | 38 | -82 | 16 | 1.68 | 3.67 |
| **Salience** |  |  |  |  |  |  |  |
| Positive association | Cortical |  |  |  |  |  |  |
|  | *Parietal* |  |  |  |  |  |  |
|  | Postcentral | R | 14 | -38 | 76 | 1.13 | 4.32 |
| **DMN –C (35)** |  |  |  |  |  |  |  |
| Positive association | Cortical |  |  |  |  |  |  |
|  | *Parietal* |  |  |  |  |  |  |
|  | Inferior | R | 54 | -38 | 52 | 1.26 | 5.72 |
| **DMN –B (06)** |  |  |  |  |  |  |  |
| Negative association | Brainstem |  |  |  |  |  |  |
|  | Vermis 9 |  | 6 | -58 | -32 | 1.08 | 5.05 |
|  |  |  |  |  |  |  |  |
